# The Catalytic Site Atlas 2.0: cataloging catalytic sites and residues identified in enzymes

**DOI:** 10.1093/nar/gkt1243

**Published:** 2013-12-05

**Authors:** Nicholas Furnham, Gemma L. Holliday, Tjaart A. P. de Beer, Julius O. B. Jacobsen, William R. Pearson, Janet M. Thornton

**Affiliations:** ^1^European Molecular Biology Laboratory, European Bioinformatics Institute, Wellcome Trust Genome Campus, Hinxton, Cambridge CB10 1SD, UK and ^2^Department of Biochemistry and Molecular Genetics, University of Virginia, 1300 Jefferson Park Ave., Charlottesville, VA 22908, USA

## Abstract

Understanding which are the catalytic residues in an enzyme and what function they perform is crucial to many biology studies, particularly those leading to new therapeutics and enzyme design. The original version of the Catalytic Site Atlas (CSA) (http://www.ebi.ac.uk/thornton-srv/databases/CSA) published in 2004, which catalogs the residues involved in enzyme catalysis in experimentally determined protein structures, had only 177 curated entries and employed a simplistic approach to expanding these annotations to homologous enzyme structures. Here we present a new version of the CSA (CSA 2.0), which greatly expands the number of both curated (968) and automatically annotated catalytic sites in enzyme structures, utilizing a new method for annotation transfer. The curated entries are used, along with the variation in residue type from the sequence comparison, to generate 3D templates of the catalytic sites, which in turn can be used to find catalytic sites in new structures. To ease the transfer of CSA annotations to other resources a new ontology has been developed: the Enzyme Mechanism Ontology, which has permitted the transfer of annotations to Mechanism, Annotation and Classification in Enzymes (MACiE) and UniProt Knowledge Base (UniProtKB) resources. The CSA database schema has been re-designed and both the CSA data and search capabilities are presented in a new modern web interface.

## INTRODUCTION

Enzymes represent ∼45% of the collective protein products of all the genomes cataloged by resources such as the UniProt Knowledge Base (UniProtKB) (1). As biological catalysts they facilitate the many metabolic processes and pathways that are critical for life to exist and have been the focus of studies by biologists and chemists for over 100 years. They are also some of the principal targets in pharmaceutical drug development, with many approved drugs acting to modify the action of enzymes implicated in disease processes. In addition they are often the focal point for biotechnology applications. Detailed information on catalytic residues and enzyme active sites are essential for understanding the relationship between protein structure and functions, design of inhibitors and enzyme design.

The Catalytic Site Atlas (CSA) (2) was established to provide curated annotations of the small number of highly conserved residues that are directly involved in undertaking the catalytic activity in enzymes whose structures have been deposited in the Protein Data Bank (PDB) (3). These curated entries can in turn be used for inferring catalytic residues in other enzyme structures through homology, using a simple PSIBlast method.

The original resource contained 177 hand-annotated entries and 2608 homologous entries, and covered ∼30% of all EC numbers found in PDB. We present here a new version of the Catalytic Site Atlas—CSA 2.0. We have significantly increased the number of curated entries to 968 and implement a new more sophisticated method for transferring the annotations to homologous structures increasing the robustness of annotation transfer. The expansion of curated entries also permits the addition of new 3D structural templates, which have been used in a revision of the Catalytic Site Search service. In addition the database schema has been re-designed, integrating it into a sister database of enzyme mechanisms: the Mechanism, Annotation and Classification in Enzymes (MACiE) database (4). We have also developed a new ontology, the Enzyme Mechanism Ontology (EMO), permitting the integration of CSA information into both MACiE and UniProtKB data structures and can be used as a controlled vocabulary for describing aspects of protein sequence and structure with chemistry and mechanistic terms across resources.

## CSA CONTENT

The principle data held in the CSA are the protein residues from experimentally determined atomic structures that are defined as catalytic. Residues are designated as being catalytic by fulfilling any one of the following criteria: (i) Direct involvement in the catalytic mechanism; (ii) Alters the pK_A_ of another residue or water molecule directly involved in the catalytic mechanism; (iii) Stabilization of a transition state or intermediate; and (iv) Activation of a substrate. Note that it does not include residues that are involved solely in ligand binding and thus differs from other resources, such as UniProtKB annotations. Entries are made with respect to the deposited PDB structure, with the potential to have many catalytic sites within a single entry.

**Figure 1.**
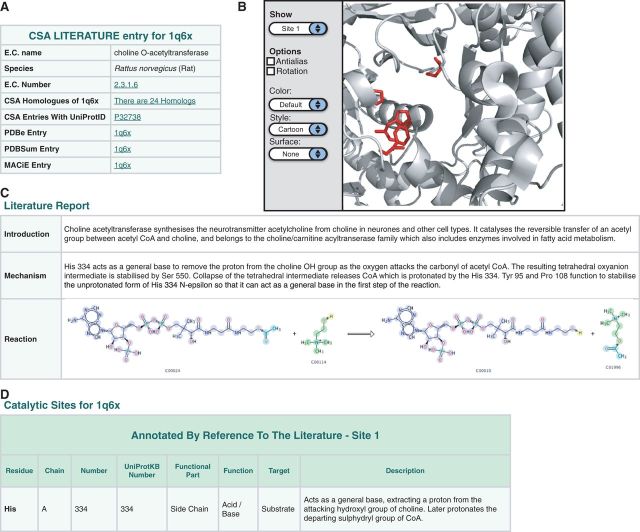
Overview of data presented for a CSA-curated entry. Meta-data descriptors such as enzyme name and species as well as internal links to find entries in the CSA that share properties along with links to external web resources are shown in a table (**A**). A 3D viewer (**B**) displays the enzyme structure, highlighting each of the catalytic sites (from a pull-down menu) in red. A free-text report of the overall reaction and mechanism are provided (**C**) with a reaction diagram marked up with groups conserved across the reaction and bond changes. (**D**) Shows the annotations held for each catalytic residues in each catalytic site.

Catalytic residue annotations are made either by manual curation or through sequence comparison. Entries to be manually annotated are chosen from the PDB based on the quality of the structure and available experimental evidence of the reaction catalysed. This includes details of the catalytic mechanism, also validated by experimental data where possible. Annotators provide a brief free-text description of the enzyme as well as a more detailed summary of the enzyme mechanism. The reaction itself is also presented and marked up to show the changes in molecular substructures and bond order/valence changes using an atom–atom matching algorithm implemented in small molecule subgraph detector (SMSD) (5). For each residue in each catalytic site the functional part of the residue is recorded as well as its function and target described using a controlled vocabulary and a short free-text description of how the residue performs the function. Evidence tags provide a direct link to the literature from which the annotations where derived. For each catalytic site a search can be performed returning all other catalytic sites in the CSA that have the same catalytic residues grouped by their E.C. numbers. In addition, hyperlinks to external resources, such as PDBSum (6) and IntEnz (7), are provided. Internal links to other entries which share the same E.C. number (8) or sequence accession numbers or PDB identifiers are made. A summary of the types of data shown for an entry is given in Figure 1.

Developers involved in the prediction of proteins of unknown function can use the extended number of curated entries to train and test the methodologies being developed. In addition individual users can access both curated and homology derived entries to gain details of the catalytic residues in a structure of interest, which has the potential to be useful in design of further experiments. The user experience has been enhanced using BioJS libraries (9) that provide a 3D viewing panel as well as a marked-up sequence viewer highlighting the catalytic residues.

**Figure 2.**
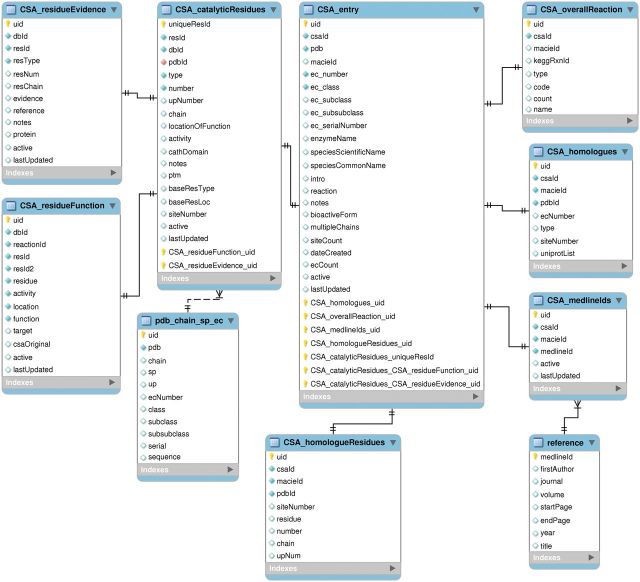
The database schema for the CSA. Relationships between tables are shown. The data are stored in a MySQL database.

As part of a wider integration of resources, the CSA has been merged with a sister database MACiE. The database schema for the CSA-related tables is shown in Figure 2. The CSA is designed as a relational database using a typical Linux, Apache, MySQL and PHP platform aided by JavaScript utilizing the BioJS library.

## INFERRING CATALYTIC RESIDUES THROUGH SEQUENCE COMPARISON

Entries are also annotated using an automated sequence comparison method that utilizes the curated entries to infer catalytic residues. 433 protein sequences from the MACIE enzyme mechanism database and the 911 sequences unique to the CSA were extracted and labeled using annotations for side-chain, main-chain, modified, reactant and spectator residues. CSA homologs in the PDB and reviewed section of UniProtKB were identified using SSEARCH36 (10) with a statistical significance threshold of E<10^−^^6^. SSEARCH alignments used the –V option to project the identity/conservative/non-conservative status of the aligned annotated functional residues from the MACiE/CSA sequences to the homologous sequences in the PDB and SwissProt.

**Figure 3.**
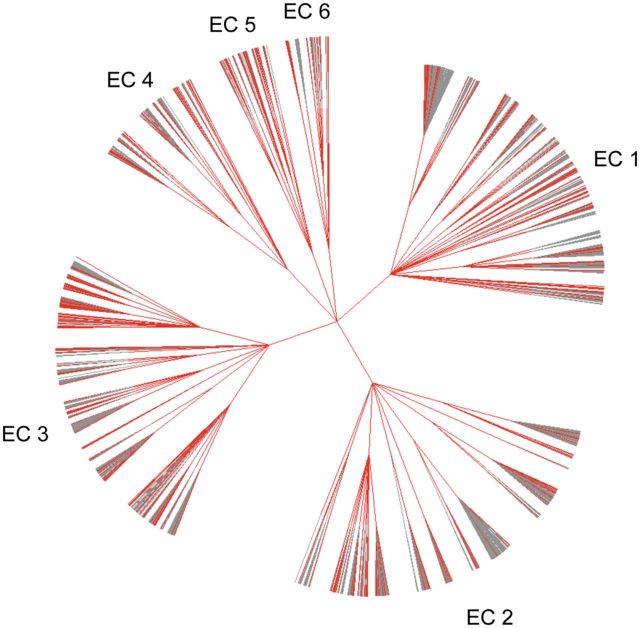
E.C. coverage in the CSA. The Enzyme Commission classification of all E.C. codes classified by the Enzyme Commission rendered as a rooted tree. Each major class is labeled with (i) Oxidoreductases, (ii) Transferases, (iii) Hydrolases, (iv) Lyases, (v) Isomerases and (vi) Ligases. Each E.C. number in the CSA is colored red, with all major classes and subclasses present and most of the sub-subclasses.

The entries derived by homology, in addition to the links to external data sources found in the curated entries, also have an internal link to the manually annotated entries that was used to infer catalytic residues. The CSA 2.0 provides a manually curated resource of 968 enzyme structures and their catalytic sites including information on the functional part of each catalytic residue and its role in the enzyme mechanism. The use of sequence comparisons extends these annotations to a further 32 216 structures annotated by homology, providing a total of 34 096 annotated structures out of possible 49 049 structures deposited in the PDB that are enzymatic. This greatly extends the 177 curated entries and 2608 entries annotated by homology in CSA 1.0. Additionally, the CSA 2.0 has entries for 1189 E.C. numbers covering all the E.C. classification classes and subclasses and most sub-subclasses (Figure 3).

## ENZYME MECHANISM ONTOLOGY

Although the CSA and MACiE resources have been developed somewhat in tandem and thus share a common data model, it is currently challenging to link these to enzyme annotations in resources such as UniProtKB due to differences in the definitions of enzyme properties and the vocabularies used in their description. Though descriptions and definitions of some of the information held in all three databases are made in existing ontologies such as GO (11) and the ChEBI (12) ontology, marrying these and applying them uniformly to all three databases proved far from trivial.

The CSA and its sister database, MACiE, utilize a controlled vocabulary, with MACiE possessing a more detailed vocabulary as it focuses on enzymes in a much greater depth to include thorough descriptions of the chemical reaction steps performed. Likewise, the reviewed section of the UniProtKB (UniProtKB/Swiss-Prot) also captures enzyme-related data at a broader protein sequence level, including information on catalytic residues. Annotations are made as both free text and using an independently developed controlled vocabulary.

To address this we have developed the EMO which builds upon the controlled vocabulary developed for MACiE and the CSA and will be submitted to the OBO Foundry (13). This vocabulary (see Supplementary Material or http://purl.bioontology.org/ontology/EMO) was created to describe the active components of the enzyme’s reactions (cofactors, amino acids and cognate ligands) and their roles in the reaction. EMO builds upon this by formalizing key concepts, and the relationships between them, necessary to define enzymes and their functions. This describes not only the general features of an enzyme, including the E.C. number (catalytic activity), 3D structure and cellular locations, but also allows for the detailed annotation of the mechanism. This mechanistic detail can be either at a gross level (overall reaction only as captured in the CSA), or the more detailed granularity of the steps and components required to effect the overall chemical transformation.

EMO allows for many different resources to be drawn together, even where annotations are only partially made, which could allow for incomplete annotation to be expanded. Communication between databases can be facilitated through the use of such a universal resource that maps disparate terms to a common data model. All annotations of CSA-curated entries have been integrated into the annotations provided in UniProtKB.

## GENERATING 3D TEMPLATES

Using the newly curated entries it is possible to build three-dimensional templates consisting of just the few atoms of the active site residues. Alternative residue types for each catalytic residue in the template can be cataloged in the template from the equivalent positions from the entries derived by homology. This extends a set of 149 templates constructed from CSA 1.0 to a total of 584 templates from CSA 2.0. The templates can be used by Jess, a fast and flexible algorithm for searching protein structures for small groups of atoms based on geometrical and chemical constraints (14), to search through new structures to find potential catalytic sites. This has been implemented in a new server CSS (http://www.ebi.ac.uk/thornton-srv/databases/CSS/). Users of this asynchronous service can either upload their own structure file or request a deposited structure (if it has not already be annotated by the CSA) to be searched using the new templates. Results are ranked based on the RMSD and a log E-value. The template made from each curated entry can be accessed from relevant the CSA entry page as well as collectively being made available for download.

## CONCLUSIONS

CSA 2.0 provides a new modern interface to a much-extended manually curated dataset of residues involved in enzyme catalytic sites and the functional role they play in the reaction. A new method for reliably extrapolating the annotations and identification of catalytic residues to homologous structures has been implemented. In addition the curated entries can be used to build 3D templates of the catalytic sites, which in turn can be used to search new structures for catalytic site identification using a revised CSS service. Furthermore a new ontology has been developed to permit the transfer of annotations relating to enzyme catalysis between resources. This has been used to include CSA annotations in UniProtKB and MACiE.

The database is available at http://www.ebi.ac.uk/thornton-srv/databases/CSA, while the CSS service can be found at http://www.ebi.ac.uk/thornton-srv/databases/CSS. Both are compatible with most modern web browsers. All the data in the CSA is downloadable and freely available to the academic community.

## SUPPLEMENTARY DATA

Supplementary Data are available at NAR Online.

## FUNDING

Wellcome Trust [081989/Z/07/A to N.F.]; European Molecular Biology Laboratory (EMBL) (to G.L.H. and various annotators); US Department of Energy Contract (in part) [DE-AC02-06CH11357 to T.A.P.deB.] as part of the Midwest Center for Structural Genomics (MCSG). Funding for open access charge: The Wellcome Trust.

*Conflict of interest statement*. None declared.

## References

[1] Uniprot Consortium (2013). Update on activities at the Universal Protein Resource (UniProt) in 2013. Nucleic Acids Res..

[2] Porter CT, Bartlett GJ, Thornton JM (2004). The Catalytic Site Atlas: a resource of catalytic sites and residues identified in enzymes using structural data. Nucleic Acids Res..

[3] Velankar S, Alhroub Y, Best C, Caboche S, Conroy MJ, Dana JM, Fernandez Montecelo MA, van Ginkel G, Golovin A, Gore SP (2012). PDBe: Protein Data Bank in Europe. Nucleic Acids Res..

[4] Holliday GL, Andreini C, Fischer JD, Rahman SA, Almonacid DE, Williams ST, Pearson WR (2012). MACiE: exploring the diversity of biochemical reactions. Nucleic Acids Res..

[5] Rahman S, Bashton M, Holliday G, Schrader R, Thornton J (2009). Small Molecule Subgraph Detector (SMSD) toolkit. J. Cheminform..

[6] Laskowski RA (2009). PDBsum new things. Nucleic Acids Res..

[7] Fleischmann A, Darsow M, Degtyarenko K, Fleischmann W, Boyce S, Axelsen KB, Bairoch A, Schomburg D, Tipton KF, Apweiler R (2004). IntEnz, the integrated relational enzyme database. Nucleic Acids Res..

[9] Gomez J, Garcia LJ, Salazar GA, Villaveces J, Gore S, Garcia A, Martin MJ, Launay G, Alcantara R, Del-Toro N (2013). BioJS: an open source JavaScript framework for biological data visualization. Bioinformatics.

[10] Sierk ML, Pearson WR (2004). Sensitivity and selectivity in protein structure comparison. Protein Sci..

[11] Blake JA, Dolan M, Drabkin H, Hill DP, Li N, Sitnikov D, Bridges S, Burgess S, Buza T, McCarthy F (2013). Gene Ontology annotations and resources. Nucleic Acids Res..

[12] Hastings J, de Matos P, Dekker A, Ennis M, Harsha B, Kale N, Muthukrishnan V, Owen G, Turner S, Williams M (2013). The ChEBI reference database and ontology for biologically relevant chemistry: enhancements for 2013. Nucleic Acids Res..

[13] Smith B, Ashburner M, Rosse C, Bard J, Bug W, Ceusters W, Goldberg LJ, Eilbeck K, Ireland A, Mungall CJ (2007). The OBO Foundry: coordinated evolution of ontologies to support biomedical data integration. Nat. Biotechnol..

[14] Barker JA, Thornton JM (2003). An algorithm for constraint-based structural template matching: application to 3D templates with statistical analysis. Bioinformatics.

